# Metabolic Syndrome in Aging Men as a Factor Affecting the Relationship between Mg, Ca, and P in Serum and Bone

**DOI:** 10.3390/ijms241310947

**Published:** 2023-06-30

**Authors:** Aleksandra Rył, Żaneta Ciosek, Aleksandra Szylińska, Alina Jurewicz, Andrzej Bohatyrewicz, Paweł Ziętek, Iwona Rotter

**Affiliations:** 1 Department of Medical Rehabilitation and Clinical Physiotherapy, Pomeranian Medical University, Żołnierska 54b, 71-210 Szczecin, Poland; zaneta.ciosek@pum.edu.pl (Ż.C.); aleksandra.szylinska@pum.edu.pl (A.S.); iwona.rotter@pum.edu.pl (I.R.); 2 Department of Specialized Nursing, Faculty of Health Sciences, Pomeranian Medical University, Żołnierska 48, 71-210 Szczecin, Poland; alina.jurewicz@pum.edu.pl; 3 Department of Orthopaedics, Traumatology and Orthopaedic Oncology, Pomeranian Medical University, Unii Lubelskiej 1, 71-252 Szczecin, Poland; bohatyrewicz@orthopedics.pl (A.B.); pawel.zietek@pum.edu.pl (P.Z.)

**Keywords:** magnesium, osteoporosis, metabolic syndrome, calcium, potassium, bones

## Abstract

The objective of this study was to evaluate the relationship between the prevalence of metabolic syndrome (MetS) and selected hormonal disorders and concentrations of magnesium (Mg), calcium (Ca), and phosphorus (P) in both blood serum and bone tissue. This study involved 152 men with and without MetS. In the blood of the patients we examined, we determined levels of: testosterone (TT), estradiol (E2), sex hormone-binding globulin (SHBG), dehydroepiandrosterone sulfate (DHEAS), insulin (I), osteocalcin (OC), and concentrations of markers of bone turnover. The concentration of Mg, Ca, and P was determined in the serum and bone tissue. In patients with MetS, the serum Ca concentration correlated with procollagen type I N-terminal propeptide (PINP) and parathyroid hormone (PTH). Among patients without MetS, the serum Ca concentration correlated with SHBG and OC, while Ca concentration in bone correlated with the lipid accumulation product (LAP) index and the body mass index (BMI). After analyzing the serum Mg concentration, positive correlations were observed with E2, PINP, and PTH in patients with MetS. In patients without MetS, the Mg concentration in bone positively correlated with the BMI and the LAP index. Our study findings suggest that increased Mg levels could have an impact on bone tissue metabolism. Elevated serum Mg levels may be associated with changes in sex hormone concentrations and alterations in bone turnover markers.

## 1. Introduction

Magnesium (Mg) is an essential mineral in the human body, alongside sodium (Na), potassium (K), and calcium (Ca). It is one of the four major cations and the second most abundant intracellular cation after potassium. By regulating the membrane gradient of these electrolytes through its influence on the sodium–potassium and Ca pumps, Mg contributes to maintaining the proper electrical potential of the cell membrane. Additionally, it enhances conductivity within the heart’s excitatory-conductive system [1].

Magnesium plays a crucial role as a trace mineral, participating in various energy-dependent transport systems, glycolysis, and oxidative energy metabolism. It has been reported to have positive effects on cholesterol levels, increasing high-density lipoprotein (HDL) and reducing low-density lipoprotein (LDL) and triglycerides (TG) by inhibiting the activity of lecithin cholesterol acyltransferase (LCAT) and HMG-CoA reductase, while increasing the activity of lipoprotein lipase (LPL) [2].

Furthermore, as a coenzyme for numerous enzymes, Mg is closely involved in carbohydrate metabolism. It exhibits mutual interactions with insulin and glucose, primarily by influencing the activity of insulin receptor tyrosine kinase and glucose transporter protein 4 (GLUT4). Thus, Mg directly contributes to the regulation of glucose translocation into the cell [3].

Some studies indicate that Mg may also have an impact on testosterone production and aid in its release for proper functioning in the body. It has been observed that higher levels of Mg can inhibit the binding of testosterone to sex hormone-binding globulin (SHBG), a transport molecule that binds to and hinders the activity of testosterone. This, in turn, increases the levels of free, bioavailable testosterone (bioT). However, despite some evidence supporting the influence of Mg on male gonadal function, the precise mechanism by which this mineral affects testosterone synthesis has not yet been fully identified [4].

Magnesium plays a vital role in bone development and mineralization. It activates bone formation by stimulating the activity of osteoblasts and acts as a cofactor for various enzymes involved in the bone formation process. Inorganic Mg compounds present in bones enhance their resilience against fractures. Besides promoting mineralization, Mg is essential for proper bone growth and facilitates efficient bone remodeling. It is worth noting that serum Mg levels are not a reliable indicator of overall Mg status, as the majority of Mg is stored in the bones. In the body, the total Mg content typically amounts to around 25 g, with 50–60% residing in the bones and soft tissues, while only 1% is found in the blood serum [5].

Magnesium deficiency is considered a risk factor for the development of metabolic syndrome (MetS), which encompasses conditions such as hyperglycemia, hypertension, hypertriglyceridemia, and insulin resistance [5]. Insufficient Mg levels can impact bone health by influencing the key regulators of Ca homeostasis, namely parathyroid hormone (PTH) and 1,25-dihydroxyvitamin D3. Hypomagnesemia can hinder the release of PTH and potentially reduce the sensitivity of target organs to circulating PTH, leading to a biochemical pattern akin to primary hypoparathyroidism [6].

The objective of this study was to evaluate the relationship between the prevalence of MetS and selected hormonal disorders and the concentrations of Mg, Ca, and P in both blood serum and bone tissue.

## 2. Results

This study investigated the relationship between anthropometric parameters, hormonal markers, markers of bone turnover, and the concentrations of Ca, P, and Mg in both serum and bone, considering the presence of MetS. Table 1 presents medians, quarters, and standard deviations of selected parameters in patients with Mets and without Mets. Significant differences were found among patients in terms of waist circumference (*p* = 0.001), BMI (*p* = 0.011), and LAP index (<0.001). When analyzing the relationship between the concentration of the analyzed parameters in patients with and without MetS, it was observed that there is no relationship between the concentration of the hormones tested and the occurrence of MetS. The only observed relationship concerned SHBG concentration (*p* = 0.047), where subjects with MetS were characterized by lower concentrations of this parameter compared to men without MetS. When analyzing the relationships between the concentrations of Mg, Ca, and P in the serum and in the bone, no statistical relationship was observed between patients with and without MetS.

Table 2 examined the correlations between the concentrations of Ca, Mg, and P in serum and bone, along with the analyzed parameters in the study. The presence or absence of MetS in patients was taken into account. The study revealed several significant correlations.

In patients with MetS, serum Ca concentration correlated with procollagen type I N-terminal propeptide (PINP) (R = 0.31 and *p* = 0.031) and PTH (R = 0.32 and *p* = 0.031). Calcium concentration in bone correlated with E2 (R = 0.35 and *p* =0.044) in patients with MetS. Among patients without MetS, serum Ca concentration correlated with SHBG (R = 0.33 and *p* = 0.011) and osteocalcin (OC) (R = 0.28 and *p* = 0.038), while Ca concentration in bone correlated with the LAP index (R = 0.47 and *p* =0.003) and BMI (R = 0.35 and *p* = 0.017).

When analyzing serum Mg concentration, positive correlations were observed with estradiol (R = 0.32 and *p* = 0.030), PINP (R = 0.42 and *p* = 0.003), and PTH (R = 0.31 and *p* = 0.037) in patients with MetS. In patients without MetS, Mg concentration in bone positively correlated with BMI (R = 0.30 and *p* = 0.042) and the LAP index (R = 0.36 and *p* = 0.026).

Furthermore, P concentration in bone showed a negative correlation with the HOMA index (R = −0.38 and *p* = 0.024) and a positive correlation with DHEAS (R = 0.42 and *p* = 0.012) in patients with MetS.

In the study, we also analyzed the relationship between the hormonal and metabolic parameters, bone turnover markers, and elements, dividing the examined men into groups taking into account the median values (as a dividing point) in serum and bone tissue (Table 3). When analyzing serum Mg concentration, a statistical relationship was established between the concentration of FT (*p* = 0.009) and bioT (*p* = 0.028) depending on the serum Mg concentration. Patients with lower serum Mg concentrations exhibited significantly higher levels of these parameters in their blood serum. It was also demonstrated that lower serum Mg concentrations were associated with lower PINP levels (*p* = 0.008) and PTH levels (*p* = 0.018). Furthermore, a relationship was observed between serum Mg concentration and serum Ca concentration (*p* < 0.001) in patients with MetS.

When analyzing blood Mg concentration above and below the median determined in the study, a relationship was identified between bone Mg concentration and Ca content (*p* < 0.001) as well as P content (*p* < 0.001) in the dry mass of bone tissue. Patients with lower bone Mg concentrations exhibited lower levels of both Ca and P in bone tissue.

## 3. Discussion

In our study, we examined 152 men (68 with MetS and 84 without MetS) who underwent total hip replacement surgery for osteoarthritis of the hip joints at the Orthopedics Clinic of the Pomeranian Medical University. Our findings revealed significant differences in waist circumference, BMI, LAP index, and SHBG concentration between the two groups. These results are consistent with the findings of Addin et al., who reported a high prevalence of abdominal obesity (62.6%) among individuals with MetS [7]. Similar studies conducted on the US and Greek populations also reported high rates of abdominal obesity, with rates of 53% and 72%, respectively [8,9]. Interestingly, countries with a high prevalence of MetS, such as Portugal (51.0%) [10], Turkey (56.8%) [11], and Tunisia (69.5%) [12], also demonstrated high rates of central obesity.

Addin et al. further confirmed a significant association between central obesity and low Mg levels (2.8%) compared to non-obese individuals (*p* = 0.041) [7]. These results are in line with the existing literature on Mg levels in patients with MetS [13,14]. The association between lower Mg levels and the aforementioned parameters suggests a potential role of adipose tissue, particularly abdominal adipose tissue, in the pathophysiology of Mg metabolism disorders. It is hypothesized that higher insulin levels in the serum of obese individuals, resulting from increased insulin resistance, lead to increased renal excretion of Mg. Consequently, lower Mg levels in the serum contribute to the development of insulin resistance.

In our own study, we observed a significant relationship between the concentrations of Mg and Ca in the serum of patients. Furthermore, when examining blood Mg levels above and below the median value determined in our study, we found a correlation between Mg levels in bone tissue and the content of Ca and P in the dry mass of bone tissue. Patients with lower Mg levels in bone tissue exhibited lower concentrations of both Ca and P.

Additionally, a study conducted on a Chinese population aimed to investigate the associations between Mg, Ca, and the Ca/Mg ratio in whole blood from patients with MetS. The study included 204 MetS patients and 204 healthy controls, matched for various factors, and aged between 48 and 89 years. The concentrations of Mg and Ca in whole blood were measured using flame atomic absorption spectrometry. Approximately 44.1% of the participants were male, with an average age of 64.0 ± 7.18 and an average body mass index of 24.3 ± 3.75. The MetS group showed significantly higher levels of Mg and lower levels of Ca and Ca/Mg ratio compared to the control group. Comparing different tertiles of Mg, the odds ratios (ORs) for MetS were found to increase from the bottom tertile (T1) to the median (T2) and top tertile (T3). Regarding Ca, T2 and T3 were negatively associated with MetS. Furthermore, an inverse relationship was observed between the Ca/Mg ratio and MetS. These findings suggest that increased Mg levels and decreased Ca levels and Ca/Mg ratio in whole blood are correlated with the presence of MetS in the Chinese adult population [15].

Park et al. also conducted a study investigating the relationship between serum Ca and Mg levels and the occurrence of MetS. The study included 213 men aged 30 to 60 years. The participants were divided into three groups based on their MetS risk score: group I (MetS risk score ≤ 1, n = 106), group II (MetS risk score = 2, n = 51), and group III (MetS risk score ≥ 3, n = 56). The study found that serum Ca levels significantly increased with higher MetS risk scores (*p* < 0.001), while no significant difference in serum Mg concentration was observed among the three groups. Subjects with high triglyceride (TG) and high blood pressure (BP) levels had higher serum Ca levels compared to those without these abnormalities. Additionally, subjects with higher glucose levels had lower serum Mg levels. The correlation analysis indicated that serum Ca was positively correlated with the MetS risk score, serum TG, and diastolic blood pressure (DBP). Conversely, serum Mg showed an inverse relationship with serum glucose. In conclusion, among middle-aged Korean men, serum Ca was positively associated with TG and BP, while serum Mg was negatively associated with serum glucose levels after adjusting for age and BMI [16].

In our own study, we also observed a significant relationship between serum Mg and Ca concentrations in patients with MetS and parathyroid hormone (PTH) levels. Magnesium deficiency leads to a reduction in 1.25(OH)2D and impairs the response of PTH. The relationship between serum PTH levels and Mg concentration appears to be complex. PTH secretion is primarily controlled by serum Ca levels, but Mg can also exert similar effects. Low Mg levels stimulate PTH secretion, while very low serum Mg concentrations induce a paradoxical blockage, resulting in clinically relevant hypocalcemia in severely Mg-deficient patients. On the other hand, PTH regulates Mg homeostasis by modulating its renal reabsorption, absorption in the gut, and release from the bone [17,18,19]. However, there is limited knowledge about the correlation between parathyroid diseases and MetS, which is why it was not possible to compare our own results with those of other authors.

In our own study, we observed a statistically significant relationship between serum Mg levels and the concentration of free testosterone and bioactive testosterone (*p* = 0.0028). This relationship was examined by dividing patients into groups based on whether their serum Mg levels were above or below the median. A similar study investigating the impact of Mg levels on testosterone concentration has been conducted by Maggio et al. who found that Mg levels were significantly and positively correlated with total testosterone. In the age-adjusted analysis, sex hormone-binding globulin (SHBG), dehydroepiandrosterone sulfate (DHEAS), and grip strength were also significant correlates of total testosterone. Additionally, body mass index (BMI) was significantly and negatively associated with total testosterone [20]. Likewise, Rotter et al. concluded that lower serum Mg levels may contribute to the development of total testosterone deficiency, arterial hypertension, diabetes, and consequently MetS [21].

Our own study results suggest a correlation between serum Mg and Ca levels in patients with MetS and parathyroid hormone (PTH) levels. Magnesium deficiency leads to a reduction in 1.25(OH)2D levels and an impaired PTH response [17,18]. The cause–effect relationship between serum PTH levels and Mg concentration appears to be complex. PTH secretion is primarily controlled by serum Ca levels, but Mg can also exert similar effects. Low Mg levels stimulate PTH secretion, while very low serum Mg concentrations can lead to a paradoxical blockage. This paradoxical blockage can cause clinically relevant hypocalcemia in severely Mg-deficient patients. On the other hand, PTH regulates Mg homeostasis by modulating its renal reabsorption, absorption in the gut, and release from the bone [19]. However, little is known about the correlation between parathyroid diseases and MetS, which is why it was not possible to compare our own results with those of other authors.

The presented study is one of the first to compare the concentrations of Mg, Ca, and P in two types of tissues—bone tissue and blood. It should be noted that bone metabolism is the result of many factors, including hormonal, metallic, and other factors. An attempt to link hormonal parameters and the accumulation of these elements is important for understanding the changes in bone tissue, where changes in concentrations are slower than in patients’ blood. An interesting result worthy of a more detailed analysis is the dependence shown in the analysis of lower and higher Mg concentrations in patients with and without MetS. It was shown here that the concentration of Mg in the bone tissue affects the concentration of Ca and P in the bone. This relationship was not observed in serum. In the light of current research on the antagonism of these elements, it is worth expanding these studies. Another result worth further research is the relationship between the concentration of male sex hormones and the accumulation of the elements in the bone. Testosterone levels appear to be related to P accumulation in bone. Our study was limited in several ways. The main limitation was that it only involved men between 60 and 75 years of age. To obtain a comprehensive view of the relationship analyzed in our study, we should have also included older patients. Another limitation is the fact that hormone levels were determined using the ELISA method, possibly leading to inaccurate measurement. It would be better to determine hormones using mass spectrometry. 

## 4. Materials and Methods

### 4.1. Study Participants

The study involved 152 men who underwent total hip replacement at Department of Orthopedics Traumatology and Musculoskeletal Oncology at Pomeranian Medical University in Szczecin, for hip joint osteoarthritis. To ensure the integrity of the study, certain exclusion criteria were applied. These included individuals with diabetes, a history of cancer, alcohol abuse, liver or kidney failure, heart failure classified as class III or IV according to the New York Heart Association (NYHA), and those taking medications that affect bone metabolism, such as mineral supplements, neuroleptics, chemotherapeutic agents, immunosuppressive drugs, corticosteroids, or antidepressants. 

### 4.2. Division into Groups

The study involved analyzing and categorizing patients based on the diagnosis of MetS. We utilized the IDF criteria for diagnosing MetS, which includes the following criteria for men: the presence of abdominal obesity (waist circumference ≥ 94 cm for European men) as a mandatory requirement, along with the coexistence of at least 2 out of the following 4 indicators: triglycerides ≥ 150 mg/dL or treatment for triglyceride metabolism disorder; HDL cholesterol < 40 mg/dL or treatment for HDL metabolism disorder; systolic blood pressure ≥ 130 mm Hg or diastolic blood pressure ≥ 85 mm Hg or treatment for hypertension; and fasting plasma glucose ≥ 100 mg/dL or treatment for type 2 diabetes.

### 4.3. Measurement of Sex Hormones and Bone Remodeling Markers

Venous blood samples were collected from all participants after an overnight fast (between 07:00 a.m. and 09:00 a.m.) and stored at −20 °C until further processing. The concentrations of total testosterone (TT, normal range for males: 2.36–9.96 ng/mL), estradiol (E2, normal range for males: 11.2–50.4 pg/mL), sex hormone-binding globulin (SHBG, normal range: 18–110 nmol/L), dehydroepiandrosterone sulfate (DHEAS, normal range: 110–470 μg/dL), and insulin (I, normal range: 5–25 μIU/mL) were determined using ELISA assays (DRG Medtek, Warsaw, Poland). Free testosterone (FT, normal range: 8.9–45.5 pg/mL) levels were calculated using the formula developed by Vermeulen: FT = (TT − N − SHBG + √((N + SHBG − TT)^2^ + 4NT))/2N, where N = 0.5217 × albumin concentration + 1 [22]. The level of bioavailable testosterone (bioT) was calculated using the formula developed by Morris at al. [23,24].

Lipid accumulation product (LAP) was calculated using the formula: LAP = (WC (cm) − 65) × TAG (mmol/L) [25]. Visceral adiposity index (VAI) was calculated according to the formula: VAI = WC (cm)/[39.68 + (1.88 × BMI)] × ((TAG/1.03) × (1.31/HDL-Ch)) [26]. Body mass index (BMI) and *waist–hip ratio* (WHR) were calculated. HOMA-IR was calculated according to the formula: fasting insulin (microU/L) x fasting glucose (nmol/L)/22.5.

The following markers of bone turnover were measured: osteocalcin (OC, normal range: 5–25 ng/mL), parathyroid hormone (PTH, normal range: 10–60 pg/mL), carboxy-terminal collagen I crosslinks (CTX-I, normal range: 0.115–0.748 ng/mL), and procollagen type I N-terminal propeptide (PINP, normal range: 85.55–2028.75 ng/mL).

### 4.4. Measurement of Serum Magnesium, Calcium, and Phosphorus

Serum and bone samples were stored at −80 °C until analysis. The levels of magnesium (Mg), calcium (Ca), and phosphorus (P) in serum were determined using inductively coupled plasma optical emission spectrometry (iCAP™ 7400 ICP-OES Analyzer; Thermo Fisher Scientific, Waltham, MA, USA). This technique is widely recognized and is powerful for the analysis and quantification of trace elements in both liquid and solid samples. Analysis was conducted in both radial and axial modes.

Serum samples were thawed at room temperature and digested using the CEM MARS 5 oven digestion system. Bone samples were also thawed at room temperature and dried overnight at 80 °C until a constant weight was achieved, after removing any adherent tissues. The bones were ground into powder using a porcelain mortar and mineralized using the CEM MARS 5 system. At least 0.1 g of bone tissue was then given a 30-min pre-reaction time in a clean hood. After the pre-reaction, 1 mL of non-stabilized 30% H_2_O_2_ was added. The samples were placed in special Teflon vessels and heated in a microwave digestion system for 35 min at 180 °C. Following digestion, the samples were removed from the microwave and allowed to cool to room temperature. Blank samples were prepared by adding concentrated nitric acid to tubes without any sample, and these blanks were subsequently diluted in the same manner. Multi-element calibration standards (ICP multi-element standard solution IV, Merck, Darmstadt, Germany) with various concentrations of inorganic elements were prepared in the same way as the blanks and samples. Deionized water (Direct Q UV, Merck, Darmstadt, Germany, approximately 18.0 MΩ) was used for the preparation of all solutions. In the calibration of bone samples, a Merck calibration standard (Darmstadt, Germany) consisting of steamed bone meal that was sieved and blended to ensure high homogeneity was used. Quality control for the determination of trace elements in serum was conducted by analyzing the SRM (8414 NIST Bovine muscle, Gaithersburg, MD, USA) using instrumental neutron activation analysis.

### 4.5. Statistical Analysis

Quantitative variables were presented as the median, standard deviation (SD), lower quartile, and upper quartile. The normality of the data was assessed using the Shapiro–Wilk test. For normally distributed data, means were compared using Student’s *t*-test. For non-normally distributed data, the nonparametric Mann–Whitney U test was used. The relationship between pairs of quantitative variables was analyzed using Pearson’s linear correlation coefficient.

The study also analyzed the relationship expressed as the ratio of the concentrations of elements in serum and bone tissue. For one analysis, participants were divided into groups based on the median values of serum and bone Mg concentrations. These median values were 24.981 mg/L for serum concentration and 3705.193 mg/kg for bone concentration.

Statistical analyses were conducted using Statistica software (version 12.0, StatSoft Poland, Cracow, Poland). The significance level was set at *p* ≤ 0.05.

## 5. Conclusions

Investigating the correlation between serum Mg and Ca concentrations in bone tissue and the occurrence of metabolic disorders in men is crucial for understanding the factors that influence the levels of these elements in tissues. Our study findings suggest that increased Mg levels could have an impact on bone tissue metabolism. Elevated serum Mg levels may be associated with changes in sex hormone concentrations and alterations in bone turnover markers. Given the widespread use of Mg supplementation among patients, further research and comprehensive investigations are necessary to delve deeper into this topic. This will contribute to a better understanding of the potential implications and benefits of Mg supplementation in the management of metabolic disorders.

## Figures and Tables

**Table 1:** The association between anthropometric parameters, hormonal markers, markers of bone turnover, and the concentrations of Ca, P, and Mg in serum and bone, categorized based on the presence of MetS.

Variable		Men with MetS *n* = 68				Men without MetS *n* = 84				*p*
		Me	Q1	Q3	SD	Me	Q1	Q3	SD	
Age [years]		67.00	62.00	70.00	5.02	65.00	63.00	70.00	5.23	0.770
WC [cm]		106.00	100.00	120.00	10.46	100.00	93.00	110.00	11.41	0.001 *
BMI [kg/m^2^]		30.45	28.69	32.60	3.87	28.57	25.56	31.37	4.06	0.011 *
WHR		1.04	0.99	1.07	0.07	1.03	0.98	1.06	0.06	0.257
VAI		3.03	2.27	4.06	1.61	1.87	1.45	2.46	1.38	<0.001 *
LAP		87.35	67.42	125.93	39.33	50.87	36.02	79.32	32.35	<0.001 *
I [µlU/mL]		10.60	6.82	16.64	13.48	9.07	6.02	14.52	10.61	0.346
HOMA-IR		2.38	1.38	3.44	7.53	1.90	1.12	2.89	2.71	0.154
TT [ng/mL]		4.24	3.30	5.70	2.27	4.62	3.74	6.10	2.03	0.163
FT [ng/mL]		0.09	0.06	0.11	0.03	0.08	0.06	0.12	0.04	0.576
bioT [ng/dL]		2.19	1.38	2.54	0.85	1.86	1.22	2.59	0.99	0.455
E2 [pg/mL]		91.75	72.15	118.30	41.78	79.26	49.10	95.90	42.02	0.064
SHBG [nmol/L]		39.32	16.69	53.38	37.85	41.78	29.20	72.37	45.83	0.047 *
DHEAS [µg/mL]		0.72	0.38	1.10	0.77	0.61	0.34	0.99	0.64	0.485
PINP [ng/mL]		852.67	558.50	1725.00	1149.91	810.17	450.68	1083.33	778.99	0.251
CTX-I [ng/mL]		0.40	0.30	0.53	0.20	0.43	0.35	0.55	0.26	0.259
PTH [pg/mL]		32.44	21.15	42.11	19.04	32.05	27.44	44.05	24.78	0.756
OC [ng/mL]		5.46	3.69	7.52	3.93	5.97	3.95	9.54	4.87	0.397
Serum concentration [mg/L]	Mg	25.62	22.38	28.15	7.49	25.35	21.91	26.75	4.38	0.296
	Ca	120.95	112.37	134.83	23.73	119.67	111.42	125.86	22.76	0.431
	P	186.03	169.52	245.49	58.54	182.95	153.28	215.80	53.52	0.223
Bone concentration [mg/kg dry weight bone]	Mg	3750.19	3270.33	4403.29	1176.83	3808.22	2773.67	4459.24	1519.66	0.731
	Ca	272,714.82	217,891.42	320,064.97	81,264.30	250,933.44	192,120.35	321,170.76	85,819.95	0.518
	P	146,036.44	125,771.38	193,627.70	66,011.00	151,539.32	127,910.86	201,348.97	81,858.88	0.801

MetS—metabolic syndrome; *n*—numbers; Me—median; Q1—lower quartile; Q3—upper quartile; SD—standard deviation; *p*—statistical significance; WC—waist circumference, BMI—body mass index; WHR—waist–hip ratio; LAP—lipid accumulation product; VAI—visceral adiposity index; I—insulin; HOMA-IR—homeostatic model assessment–insulin resistance; TT—total testosterone; FT—free testosterone; E2—estradiol; SHBG—sex hormone-binding globulin; DHEAS—dehydroepiandrosterone sulfate; PINP—procollagen type I N-terminal propeptide; CTX-I—carboxy-terminal collagen I crosslinks; PTH—parathyroid hormone; and OC—osteocalcin. * Statistically significant parameter.

**Table 2:** Selected correlations between the concentrations of Ca, Mg, and P in serum and bone, and the analyzed parameters in the study.

Variable			Men with MetS *n* = 68		Men without MetS *n* = 84	
			R	*p*	R	*p*
Serum concentration	Ca	FT	−0.15	0.343	−0.25	0.076
		BioT	−0.13	0.393	−0.21	0.137
		PINP	0.31	0.031 *	0.14	0.301
		SHBG	0.21	0.152	0.33	0.011 *
		PTH	0.32	0.031 *	0.17	0.207
		OC	−0.21	0.155	0.28	0.038 *
	Mg	FT	−0.25	0.099	−0.18	0.202
		BioT	−0.23	0.125	−0.15	0.304
		E2	0.32	0.030 *	0.25	0.067
		PINP	0.42	0.003 *	0.23	0.093
		SHBG	0.28	0.059	0.26	0.051
		PTH	0.31	0.037 *	0.36	0.007 *
	P	FT	−0.05	0.738	−0.27	0.053
		BioT	−0.03	0.849	−0.29	0.038 *
Bone concentration	Ca	BMI	0.10	0.557	0.35	0.017 *
		WHR	0.23	0.179	0.24	0.109
		VAI	0.38	0.026 *	0.20	0.233
		LAP	0.30	0.082	0.47	0.003 *
		E2	0.35	0.044 *	0.12	0.441
		PINP	0.10	0.567	0.11	0.495
		SHBG	0.17	0.311	−0.35	0.021 *
	Mg	BMI	0.19	0.256	0.30	0.042 *
		WHR	0.31	0.071	0.19	0.203
		VAI	0.25	0.154	0.09	0.590
		LAP	0.32	0.058	0.36	0.026 *
	P	WHR	0.15	0.396	0.31	0.038 *
		FT	−0.16	0.374	−0.28	0.086
		HOMA-IR	−0.38	0.024 *	0.03	0.873
		BioT	−0.14	0.440	−0.27	0.100
		DHEAS	0.42	0.012 *	−0.23	0.136

MetS—metabolic syndrome; *n*—numbers; R—correlation coefficient; *p*—statistical significance; BMI—body mass index; WHR—waist-hip ratio; LAP—lipid accumulation product; VAI—visceral adiposity index; *HOMA-IR*—Homeostatic Model Assessment–Insulin Resistance; FT—free testosterone; E2—estradiol; SHBG—sex hormone-binding globulin; DHEAS—dehydroepiandrosterone sulfate; PINP—procollagen type I N-terminal propeptide; PTH—parathyroid hormone; and OC—osteocalcin. * Statistically significant parameter.

**Table 3:** Relationships stratified by patients above and below the median serum Mg concentration and median blood analysis concentration.

Variable		Patients with Serum Mg below the Median, *n* = 70			Patients with Serum Mg above the Median, *n* = 82			*p*	Patients with Bone Mg below the Median, *n* = 77			Patients with Bone Mg above the Median, *n* = 75			*p*
		Me	Q1	Q3	Me	Q1	Q3		Me	Q1	Q3	Me	Q1	Q3	
Age [years]		67.00	63.00	70.50	65.00	62.00	70.00	0.194	65.00	63.00	68.00	66.00	61.00	70.00	0.454
WC [cm]		103.00	98.00	114.00	102.00	96.50	112.00	0.510	102.00	97.00	111.00	106.50	99.00	114.50	0.172
BMI [kg/m^2^]		29.06	26.57	32.60	29.55	27.73	31.64	0.896	29.22	26.81	31.38	30.22	27.73	32.60	0.217
WHR		1.01	0.98	1.05	1.03	0.99	1.07	0.292	1.00	0.96	1.06	1.03	0.98	1.07	0.158
VAI		2.26	1.86	3.46	2.35	1.54	3.43	0.633	2.11	1.63	3.11	2.41	1.71	4.08	0.300
LAP		67.42	47.91	94.17	68.88	45.44	102.07	0.913	51.79	44.81	87.81	76.21	52.09	94.98	0.061
HOMA-IR		1.94	1.27	3.32	1.94	1.26	3.20	0.863	1.82	1.24	3.15	1.92	1.25	2.69	0.857
TT [ng/mL]		4.15	3.39	6.01	4.30	3.32	5.59	0.710	4.76	3.42	6.33	4.27	3.44	5.43	0.254
FT [ng/mL]		0.09	0.07	0.12	0.07	0.04	0.11	0.009 *	0.09	0.06	0.12	0.09	0.05	0.11	0.257
bioT [ng/dL]		2.12	1.48	2.49	1.62	1.05	2.49	0.028 *	1.91	1.44	2.65	1.96	1.11	2.47	0.331
E2 [pg/mL]		73.36	43.46	100.43	81.48	58.16	115.72	0.068	81.48	43.07	106.22	81.03	63.91	109.50	0.391
PINP [ng/mL]		674.91	165.82	891.00	857.67	515.82	1397.64	0.008 *	827.67	219.45	1101.00	832.67	612.67	1041.00	0.823
SHB [nmol/L]		38.05	18.57	62.98	44.46	27.08	70.20	0.075	40.17	21.00	63.78	40.14	19.21	69.26	0.898
DHEAS [µg/mL]		0.62	0.36	1.03	0.68	0.35	1.00	0.667	0.76	0.33	1.21	0.72	0.36	0.87	0.596
CTX-I [ng/mL]		0.38	0.31	0.53	0.42	0.31	0.53	0.752	0.40	0.32	0.49	0.40	0.29	0.58	0.798
PTH [pg/mL]		29.13	19.19	40.20	36.48	27.30	46.15	0.018 *	32.11	19.46	43.73	34.53	25.14	42.60	0.518
OC [ng/mL]		5.87	3.95	7.62	5.43	3.36	8.41	0.569	5.23	3.88	7.08	5.63	3.58	8.62	0.806
Serum concentration [mg/L]	Mg	21.94	20.79	23.70	27.37	26.00	28.91	<0.001 *	23.66	21.00	26.34	24.47	22.62	27.58	0.225
	Ca	114.93	105.04	122.10	125.87	118.88	136.39	<0.001 *	117.55	108.69	123.98	121.93	111.91	126.49	0.276
	P	194.85	168.22	241.24	182.86	154.66	210.07	0.115	188.16	162.66	232.57	187.34	166.96	232.66	0.892
Bone concentration [mg/kg]	Mg	3461.50	2474.19	4390.75	3679.07	3106.69	4421.61	0.399	2749.03	2169.25	3300.97	4443.07	4110.75	5193.62	<0.001 *
	Ca	247,410.06	184,755.26	318,564.87	250,933.44	199,607.43	302,388.25	0.932	207,257.22	154,508.66	245,303.57	312,614.67	276,367.54	351,399.86	<0.001 *
	P	146,706.42	123,116.14	190,721.26	160,905.78	125,546.72	199,137.57	0.347	139,816.19	105,196.08	162,624.56	193,719.65	144,154.31	260,890.97	<0.001 *

MetS—metabolic syndrome; *n*—numbers; Me—median; Q1—lower quartile; Q3—upper quartile; SD—standard deviation; *p*—statistical significance; WC—waist circumference; BMI—body mass index; WHR—waist–hip ratio; LAP—lipid accumulation product; VAI—visceral adiposity index; I—insulin; HOMA-IR—homeostatic model assessment–insulin resistance; TT—total testosterone; FT—free testosterone; E2—estradiol; SHBG—sex hormone-binding globulin; DHEAS—dehydroepiandrosterone sulfate; PINP—procollagen type I N-terminal propeptide; CTX-I—carboxy-terminal collagen I crosslinks; PTH—parathyroid hormone; and OC—osteocalcin. * Statistically significant parameter.
